# Exploring the Rating of Perceived Exertion in the First Repetition (RPE-1) on Post-Activation Performance Enhancement in Trained Individuals: A Pilot Study

**DOI:** 10.3390/sports13060183

**Published:** 2025-06-11

**Authors:** Iván Chulvi-Medrano, Fernando Martín, Javier Gene-Morales, Álvaro Juesas, Pablo Jiménez-Martínez, Juan C. Colado

**Affiliations:** 1 Research Group on Prevention and Health in Exercise and Sport (PHES), Department of Physical and Sports Education, University of Valencia, 46010 Valencia, Spain; ivan.chulvi@uv.es (I.C.-M.); fernando.martin-rivera@uv.es (F.M.); alvaro.juesastorres@uchceu.es (Á.J.); juan.colado@uv.es (J.C.C.); 2 Department of Education, University CEU Cardenal Herrera, 12006 Castellón, Spain; 3 ICEN Institute, 28840 Madrid, Spain

**Keywords:** variable resistance training, strength training, rate of perceived effort, elastic bands, neuromuscular activation, potentiation

## Abstract

(1) Background: Limited research has examined elastic resistance preconditioning effects on post-activation performance enhancement (PAPE) using the rate of perceived exertion (RPE) as an intensity indicator. This pilot study aimed to evaluate the application of RPE to the elastic resistance push-press (ERPP) exercise performed at maximum velocity for its effects on PAPE. (2) Methods: Twenty-four trained, healthy young adults participated in this study and performed both a conventional warm-up and a warm-up combined with six repetitions of an elastic resistance push-press (ERPP) at 85% 1RM/RPE of the first repetition (RPE-1) of 6 out of 10. The pre-post variables assessed were push-ups, a countermovement jump (CMJ), and 10 and 20 m sprints. A repeated-measures ANOVA was conducted. Additionally, between-subject variability was adjusted for individual performance. (3) Results: In the push-up, a significant improvement (*p* ≤ 0.05) of 20.41% in mean propulsive velocity was observed among the less strong participants. A medium effect size improvement (d = 0.47; *p* = 0.13) was observed in CMJ performance (7.93%) among the less strong participants. Significant improvements were noted in sprint performance over 10 m (8.07%) and 20 m (6.23%) in the ERPP group compared with the standard warm-up, particularly in the less strong participants. The stronger participants exhibited no significant changes in either variable. (4) Conclusions: We concluded that ERPP effectively induced PAPE in the less strong participants. Additionally, RPE-1 is proposed as a tool to monitor intensity in elastic band resistance to induce PAPE.

## 1. Introduction

Post-activation performance enhancement (PAPE) is a physiological phenomenon that describes the enhancement in performance following high-intensity specific conditioning activities [1]. Studies have demonstrated that athletes can achieve significant improvements in power and speed by employing techniques to induce PAPE, which are mediated by several plausible mechanisms, including increased muscle temperature, enhanced muscle fiber recruitment, and improved metabolic readiness [1,2]. These transient changes enhance muscle activation, thereby increasing the capacity to generate force, with important implications for optimizing performance in sports that require high-intensity efforts [1,3].

The phenomenon of PAPE has been extensively studied across various conditions, including, but not limited to, maximal dynamic actions [4,5], isometric actions [6], and the use of Yo-Yo devices [7], as well as the application of electrostimulation and vibratory stimuli [8]. Consequently, the role of newer specific interventions, such as elastic resistance, in modulating PAPE requires further investigation to establish evidence-based guidelines for practitioners.

Elastic band resistance training has emerged as a versatile and effective method to improve athletic performance. Elastic bands provide variable resistance, which can be adjusted to target different muscle groups and physiological responses, as well as accommodate various fitness levels [9,10]. The benefits of resistance band training include increased muscle strength, improved flexibility, and enhanced neuromuscular coordination [11,12], which enhance whole-body strength and performance across the lifespan [11]. This type of resistance offers characteristics that make it particularly suitable for warm-up purposes, such as user-friendliness, portability, and cost-effectiveness [11]. A few studies aimed at the acute effects of PAPE, including elastic resistance, with positive results in sprint [13,14] and jump performance [14,15]. Recent evidence suggests that variable or accommodated resistance provided by elastic bands may also enhance the PAPE effect. In a study by Masel and Maciejczyk [16], the synergistic application of elastic bands and free weights was investigated, revealing an improved PAPE 90 s after performing a conventional deadlift at 80% of one repetition maximum for three repetitions. The authors suggested that incorporating accommodated resistance is effective for achieving PAPE in explosive activities following a brief interval [16]. The selection of the resting period between the inducing activity and PAPE assessment was informed by findings from a prior study, which examined the effects at intervals of 90 s, 120 s, and 150 s, concluding that 90 s was the optimal duration to enhance jump performance through PAPE [17].

Another study examined the effects of performing a half-squat with and without elastic overload to induce PAPE in young physical education students. The participants executed the half-squat exercise using elastic bands at two intensities: three-repetition maximum and five-repetition maximum. This was performed to assess the generation of PAPE in jump, sprint, and change of direction abilities at 15 s, 4 min, and 8 min post-exposure. The results indicated that both intensities effectively generated PAPE across all variables at 4 min post-exposure [18].

The only study that employed elastic stimulation in the upper extremities to describe a PAPE phenomenon was conducted by [19]. The participants underwent an intervention involving a vertical pulling movement using “superbands”, with a load of three sets of three repetitions, applying a perceived exertion scale (RPE) ranging from moderate to hard, which enabled the participants to perform additional repetitions in the pull-up exercise. After this stimulus, the subjects were tested by performing pull-ups following an 8 min interval after an intervention using elastic bands. The authors registered an increase in the number of repetitions in the pull-up test and concluded that the use of moderate- and high-intensity elastic resistance training represents an adequate stimulus for inducing PAPE in the upper extremities.

Since the development of Borg’s Rating of Perceived Exertion (RPE) scale [20], it has been widely utilized and adapted across various exercise modalities [21], including cardiovascular training [22], resistance training [23], and resistance training with elastic bands [24]. Recently, it was proposed that RPE in the first repetition serves as a significant and reliable indicator, correlating with the total repetitions completed at each RPE level by trained individuals performing resistance training with elastic bands at both moderate and maximal intentional velocities [25,26]. Although RPE has been extensively studied across conventional exercise modalities, there is still limited research exploring its application in interventions designed to induce post-activation performance enhancement (PAPE). To the best of our knowledge, no study has investigated the potential of performing full-body exercise using elastic bands to induce PAPE, monitoring the intensity by applying RPE-1 [25]. The RPE-1 scale, typically ranging from 1 (very easy) to 10 (maximal exertion), provides a subjective measure of the intensity perceived by the individual at the onset of the set [25]. Therefore, a full-body exercise involving an elastic resistance push-press (ERPP), monitored at RPE-1, was selected as a conditioning stimulus to investigate its effects on PAPE.

The aim of the present study was to analyze whether performing the push-press exercise with elastic resistance at maximal intentional velocity and a high resistance percentage, as monitored by RPE-1, induces a transient increase in push-up speed, jump performance, and sprint ability. The initial hypothesis posited that ERPP would improve performance in all tests.

## 2. Materials and Methods

### 2.1. Participants and Ethical Considerations

Voluntary participants from the University of Valencia were recruited through appeals on social networks, posters in the faculty, and direct advertisement to the general community. All the participants were fully informed about the study procedures and provided written consent prior to their involvement. All the participants met all the inclusion criteria: (i) men and women aged 18 to 35 years and (ii) strength training experience of at least 6 months. The subjects agreed to participate in this study voluntarily and, after complying with all the indications and requirements listed above, signed an informed consent form.

The sample consisted of twenty-four healthy, trained participants (age 23 ± 3 years; height 178.83 ± 8.57 cm; weight 78.10 ± 12.27 kg).

### 2.2. Study Design

**Figure 1:**
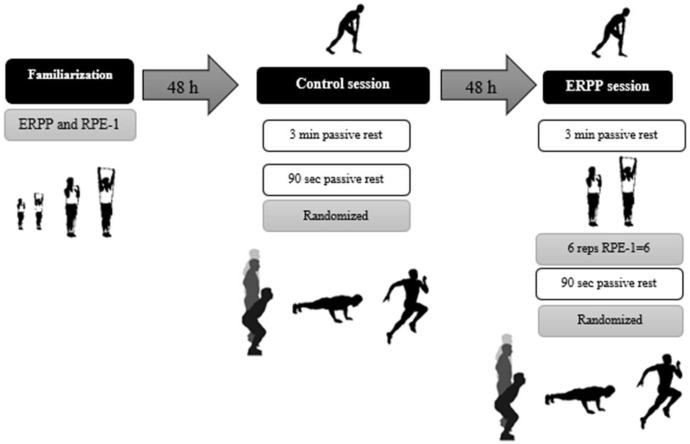
Schematic overview of the experimental design. ERPP: elastic resistance push-press; RPE: rate of perceived exertion.

The present pilot study was conducted using a descriptive design. The voluntary participants from the Faculty of Physical Activity and Sport Sciences at the University of Valencia were randomly assigned to complete two experimental conditions (Figure 1): (a) a standard warm-up and (b) ERPP at maximal speed intention with a load based on a perceived exertion rate of 6 on the first repetition, corresponding to approximately 85% of 1RM [25,26].

A previous familiarization phase was conducted, during which the participants were informed about the upcoming sessions and the tests they would perform (warm-up, PAPE test, push-up performance measurement, and familiarization with perceived exertion). After the familiarization session (minimum 24 h), the participants underwent two experimental sessions interspersed for at least 48 h: a control session performed a conventional warm-up and an experimental session that conducted the same warm-up, but after a 3 min rest, they performed an elastic resistance push-press (ERPP) for 6 repetitions at 85% 1RM/RPE-1 of 6 out of 10.

The effect of PAPE was assessed through tests of maximum velocity push-ups, counter-movement jumps (CMJs), and sprinting performance at 10 and 20 m after the experimental procedures. To ensure standardization, both groups in the experimental sessions were instructed to rest for 90 s: the conventional group rested after a 3 min pause before testing, while the ERPP group rested following the completion of the PAPE protocol.

The participants were required to meet the following conditions prior to the beginning of the sessions: not having consumed any stimulant food supplements (e.g., caffeine) in the hours leading up to the session; not having engaged in strenuous physical activity during the 24 h prior to this study; and having obtained at least eight hours of sleep the night before to ensure adequate rest.

#### 2.2.1. Familiarization Stage

During the initial part of the familiarization visit, the experimental procedures were explained, and the participants practiced the standard warm-up that would be performed at the start of each session and during the assessment tests. The participants were introduced to the Rating of Perceived Exertion (RPE) scale, with a brief explanation of its general applications and the specific context required for this study [25,26]. They were instructed that the scale can assess overall body effort or active muscles and can be applied to either the first or last repetition. In this case, the participants were asked to focus on the active muscles’ perceived exertion during the first repetition rather than the overall tension in the arms or legs. The familiarization process for RPE-1 was as follows: (i) The participants first performed a repetition at maximum intended speed without an elastic band to establish what an RPE-1 of 0 felt like. (ii) Once the participants understood what an RPE-1 of 1 felt like, they were introduced to an RPE-1 of 10, performed maximal isometric effort, where they could not move the load despite exerting maximal effort. This helped the participants grasp the sensation of an RPE-1 of 10. (iii) Next, the participants experienced an RPE-1 of 5 by performing a repetition with a band providing moderate resistance, after which they were asked to rate their perceived exertion. Following this, the participants completed repetitions at different resistance levels to further refine their understanding of RPE-1 values, progressing to an RPE-1 of 6. The participants were instructed to indicate RPE-1 based on the resistance of the band they were using. A THERABAND high-resistance bands model (THERABAND, Hygenic Corporation, Akron, OH, USA) was used and identified based on the original color. The elastic band color corresponding to RPE-1 6 was registered for the experimental session.

#### 2.2.2. Intervention

(a) Conventional Warm-Up

The conventional warm-up consisted of two laps of a basketball court at a light jog. This was followed by stretching and dynamic mobility exercises at 10 repetitions each (alternating lunges, squats in both directions, and jumps with one-legged reception; front and lateral hip mobility by lifting the legs forward and back; and three repetitions of skipping on the spot with full speed exit at maximum speed up to 5 m). Finally, the subjects conducted five squats, five push-ups, and five burpees at intentional maximum velocity.

(b) Elastic Resistance Push-Press (ERPP)

**Figure 2:**
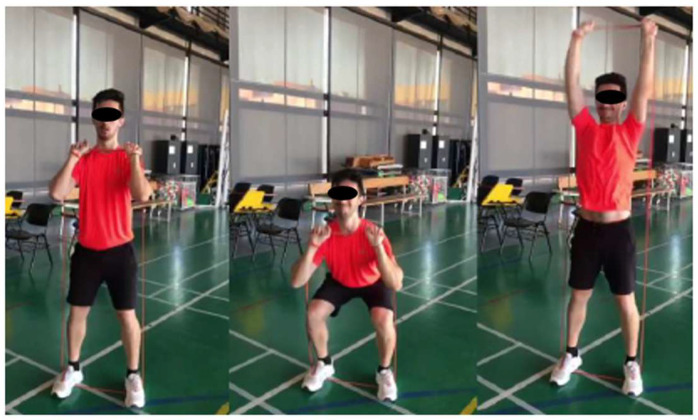
Elastic resistance push-press (ERPP).

The push-press is a global exercise that involves the lower and upper limbs in explosive movement. The subjects were instructed to perform the correct technique that was described previously by the National Strength and Conditioning Association [27] (Figure 2). In push-press exercise execution, speed is a critical factor, as the rapid activation of muscles and their precise coordination are necessary to generate maximum force in the shortest possible time [28].

Additionally, it has been suggested that high-velocity elastic resistance band training is more effective than free-weight training in specific phases of execution because of the adaptations it can induce, showing a positive impact on sprint speed [29]. In the ERPP session, after performing the conventional warm-up, the subjects were instructed to rest in a sitting position for 3 min. Following the rest period, ERPP was performed using an elastic band that was registered in the familiarization phases as RPE-1 = 6.

To create resistance, THERABAND high-resistance bands (THERABAND, Hygenic Corporation, Akron, OH, USA) of varying resistances were used in this study. The levels of estimated resistance based on the pull forces required to stretch the bands to 100% and 200% can be found in the corresponding documentation provided by THERABAND.

### 2.3. Testing Procedures and Instrumentations

#### 2.3.1. Push-Up Test

**Figure 3:**
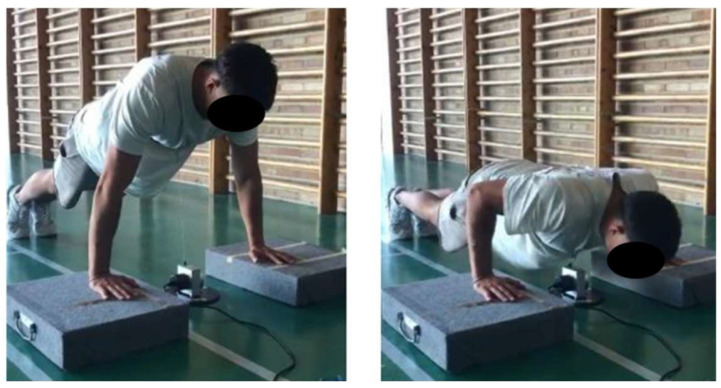
Push-up test.

The participants were instructed to perform three repetitions at maximum speed in a push-up exercise, lowering to a 90° elbow flexion angle, and positioning the hands at a 150% biacromial distance. This approach allowed for the assessment of maximum velocity during both the concentric and eccentric phases of each repetition. The encoder was securely attached to the participants at chest level (at the level of the body of the sternum) using a belt to ensure proper positioning (Figure 3).

To maintain accuracy during the measurements, the participants placed their hands on plates to prevent their chests from contacting the linear encoder (Chronojump Boscosystem, Barcelona, Spain) throughout the movement, which could lead to measurement errors. Additionally, before commencing each measurement, the encoder was positioned to ensure that the movement was as linear as possible relative to the encoder. These procedures were conducted in accordance with the protocol established by Saez-Berlanga et al. [30].

The dependent variables considered in this study were maximum velocity (Vmax) and mean propulsive velocity (MPV). These variables were selected as they most effectively represent the explosiveness and power of a movement [31], particularly within a protocol focused on enhancement and execution speed. From each measurement, the repetition performed at the highest velocity was selected for the analysis based on these variables.

#### 2.3.2. Countermovement Jump (CMJ)

**Figure 4:**
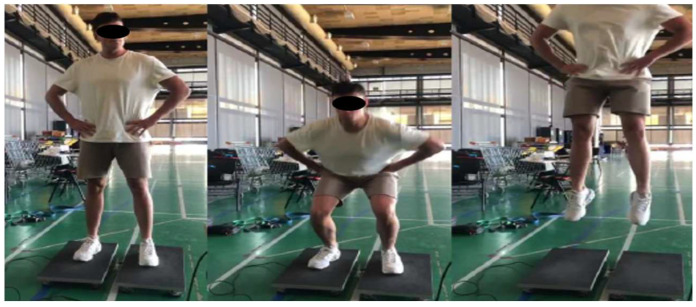
Countermovement jump.

The subjects performed 3 maximal countermovement jumps. The subjects were asked to start the movement in an upright standing position with their hands on their hips. When they were ready, they dropped to volitional depth and jumped as high as possible. All jumps were performed with their hands held on their hips to limit any upper extremity influence on the jumps. CMJ performance was analyzed using a calibrated force plate (Forcedecks 4000, Vald Performance, VIC, Australia) (Figure 4).

#### 2.3.3. Sprint: 10 m and 20 m

The linear sprint test was used to monitor the sprint performance of the subjects before and after the intervention. Measurements were conducted using high-precision photocells (Fitlight Trainer, ON, Canada) placed at the starting line and distances of 10 and 20 m, respectively. The starting area was marked on the ground. The participants were instructed to respond to an auditory signal provided by a member of the research team to initiate the sprint. Upon receiving this signal, the timing of the sprint trial was recorded by the photocells. The subjects were verbally encouraged to perform their best. The results are expressed in meters per second.

### 2.4. Statistical Analysis

Statistical analysis was performed using SPSS software (version 28.0.1.1; IBM Corp., Armonk, NY, USA). The Shapiro–Wilk test was applied to assess the normality of the two dependent variables. The data are presented as the mean values and standard deviations.

A one-way repeated-measures ANOVA was conducted to examine the potential effects on mean propulsive velocity and peak velocity when applying a post-activation performance enhancement (PAPE) technique through elastic resistance push-press exercises compared to a standard warm-up. Based on the descriptive values, an additional inter-subject factor was included in the repeated measures ANOVA to account for the participants’ maximal strength, as assessed by the median of bodyweight push-up performance (<203 N or >203 N). In the case of CMJ and sprint tests, jump performance was considered as the inter-subject factor (<37 cm or >37 cm) because previous data supported the correlations between jump and sprint performance [32,33,34]. All cases met Mauchly’s test of sphericity assumption.

The effect size was calculated using partial eta squared (ƞp^2^), derived from the ANOVA and interpreted as follows: small effect ≥0.01; medium ≥0.06; and large ≥0.14. Post hoc comparisons were performed using least significant difference (LSD) adjustments. The effect size for the post hoc comparisons between conditions was calculated using Cohen’s d to quantify the practical magnitude of the observed differences between the conditions. This analysis complemented the statistical results and allowed for the interpretation of the clinical or practical relevance of the effects found. The interpretation of Cohen’s d effect sizes was classified as follows: d = 0.2, small effect; d = 0.5, medium effect; and d = 0.8, large effect [35].

Statistical significance was set at *p* ≤ 0.05. Because of the exploratory nature of this pilot study and the small sample size, trends toward significance or marginal significance were considered for *p*-values between 0.06 and 0.15, always interpreted in conjunction with a moderate or large effect size, as well as the practical or applied relevance of the findings.

## 3. Results

The results are presented as the mean and standard deviation. The subjects’ characteristics were 23.00 (3.02) years, a height of 179.08 (9.00) cm, and a weight of 77.26 (11.88) kg.

### 3.1. Push-Up

Overall, the statistical ANOVA analysis revealed any condition x time interaction for MPV: F_[1,22]_ = 2.23, *p* = 0.14, np^2^ = 0.09 or Vmax: F_[1,22]_ = 0.14, *p* = 0.71, np^2^ = 0.01. However, when considering the participants’ strength levels, a significant trend was observed in the conditions for MPV: F_[1,22]_ = 2.52, *p* = 0.12, np^2^ = 0.10 but not for Vmax: F_[1,22]_ = 1.57, *p* = 0.22, np^2^ = 0.07. ERPP appeared to generate greater MPV in the weaker participants. This finding was confirmed by a post hoc analysis using the least significant difference (LSD) test (*p* = 0.04), which showed an increase of approximately 20.41% in the ERPP condition compared with the standard warm-up among the weaker participants (Table 1). The maximum velocity in the weaker subjects changed significantly (*p* = 0.26) or higher (*p* = 0.54).

### 3.2. CMJ

The jump performance overall interaction showed no significant differences in the interaction condition × time: F_[2,44]_ = 0.32, *p* = 0.73, np^2^ = 0.01. After adjusting for baseline jump performance (Table 2), a trend towards statistical significance was observed in the weaker participants, with a 7.93% improvement in performance, accompanied by a moderate effect size: F_[2,44]_ = 2.06, *p* = 0.13, np^2^ = 0.09.

### 3.3. Sprint

An overall ANOVA interaction was found for sprint performance by intervention: F_[2,44]_ = 752.27, *p* ≤ 0.01, np^2^ = 0.97. Table 3 shows the mean values and standard deviations of sprint performance. PAPE was significantly more effective: F_[2,44]_ = 3.83, *p* = 0.03, np^2^ = 0.15. for the ERPP less strong participants. Specifically, for this participant profile, at the 10 m distance, performance improved by 8.07%, and at the 20 m distance, the improvement was 6.23%.

It is also noteworthy that among the stronger participants, regardless of the distance, the ERPP protocol appeared to significantly reduce (*p* ≤ 0.05) their velocity by 5.70% and 5.18%, respectively.

## 4. Discussion

The main purpose of this study was to examine whether incorporating a conventional warm-up push-press exercise with elastic resistance performed at maximal intentional velocity leads to a temporary enhancement in push-up execution speed, jump performance, and sprint ability. The main finding was that ERPP induced PAPE in less strong participants, improving MPV in push-ups and enhancing CMJ and sprint performance over 10 and 20 m, in line with previous data [36]. The novelty of our study lies in the use of RPE-1, which may allow for better intensity adjustments to reduce the risk of fatigue during actions that induce PAPE. This approach aims to balance conditioning activities and the contraction history for potentiation, ensuring they induce PAPE without leading to fatigue [1,37].

Our data are inconsistent with previous observations, indicating that this effect is more pronounced in individuals with greater levels of muscle strength compared to those with lower strength levels [36,38]. We speculated that this phenomenon may be attributed to the need for a longer familiarization period to achieve a more accurate RPE-1. Since stronger individuals can generate greater force, failing to reach the necessary thresholds may avoid the induction of PAPE.

The effect of PAPE has been primarily analyzed in the lower limbs, with less information available on PAPE in the upper limbs. Additionally, many studies have focused on measuring the effect of PAPE on throwing performance [39,40]. Our study analyzed execution speed during the push-up exercise. This method was previously applied to the bench press exercise by Tsoukos et al., who showed that a 10% velocity loss (from the first repetition in a set) used during the bench press as a PAPE induction activity led to a significant increase in MPV during the subsequent performance test among resistance-trained males [41,42]. Our results did not reach a significant condition × time interaction for either mean propulsive velocity (MPV) or maximum velocity (Vmax); however, a trend toward significance was observed for MPV when the participants were stratified by strength level, as indicated by a 20.41% increase in MPV without affecting Vmax. This increase yielded a moderate effect size (ηp^2^ = 0.10). Unfortunately, we did not measure fatigue, making it impossible to compare our findings with those reported by Tsoukos’ group.

Recently, it was suggested that a PAPE effect can be achieved in the upper limbs regardless of whether the agonist or antagonist musculature is conditioned [43]. In this context, it is relevant to note that our data indicate a PAPE effect, similar to findings by [19], who observed an effect of PAPE on pull-up performance 8 min after an intervention with resistance bands. Their protocol involved vertical pulling movements with a load of three sets of three repetitions using a perceived exertion (RPE) scale from moderate to hard, which resulted in an increased number of pull-up repetitions. Methodologically, there are differences, such as in the use of RPE. In the case of Vargas-Molina et al.’s study [19], RPE was measured at the end of the set, whereas in our study, we used RPE-1.

Similar to the push-up results, the overall interaction for jump performance showed no significant differences between condition and time (*p* > 0.05, ηp^2^ = 0.01). However, despite not reaching statistical significance, the observed trend toward significance among the weaker participants, along with its medium effect size and 7.93% improvement in CMJ performance, may reflect a meaningful physiological response, particularly relevant in applied settings. These findings warrant further exploration with larger sample sizes to confirm the observed trend, especially considering that the use of resistance bands to generate accommodating resistance has been shown to be effective in inducing PAPE in previous studies. For instance, the recent synergistic application of free weights and resistance bands has also been investigated, showing enhanced PAPE 90 s after performing conventional deadlifts for three repetitions at 80% of one-repetition maximum [16].

The exclusive use of resistance bands to induce PAPE in jump capacity has been explored in several studies. In one such study, Peng et al. [18] compared the effects of half-squats, with and without elastic overload, on jump performance in young physical education students. The participants performed half-squats using resistance bands at two intensities (3 RM and 5 RM) to examine whether these conditions could induce PAPE in jumps at 15 s, 4 min, and 8 min post-intervention. The results suggested that both intensities successfully generated PAPE in all variables at 4 min post-exposure. In our study, the intensity used was 85%, corresponding to 6RM, and this could explain the difference in the results.

Regarding sprint performance, our data are aligned with those of Peng et al. [18], who found that performing exercises with elastic overload improved sprint capacity. The ERPP condition shows better times in the sprint test compared to the conventional warm-up, regardless of strength levels. Our results show that the sprint performance of the stronger subjects was improved by 8.07% and by 6.23% at 10 m and 20 m, respectively. Conversely, among the stronger participants, the ERPP protocol significantly reduced velocity by 5.70% and 5.18% at 10 and 20 m, respectively. This suggests that the protocol may benefit weaker athletes but could potentially impair performance in stronger individuals.

Combined exercise with resistance bands has been shown to be an effective strategy to induce PAPE in sprint performance [13,14]. However, unfortunately, we did not find any studies that have exclusively used elastic resistance to induce PAPE.

In all the discussed papers, there is a different methodological issue regarding the time elapsed since the PAPE-inducing activity and the test. This is a critical issue, as performance increases typically occur between 6 and 10 min post-inductive activity. However, research has shown that PAPE effects can be observed with intervals shorter than 3 min, suggesting different mechanisms of action, such as an increase in motor unit recruitment and a change in muscle fiber pennation angle [3]. One possible mechanism is that resistance training provides distinct advantages, including progressive loading and dynamic muscle recruitment, which cannot be entirely replicated by traditional free weights. In our study, we selected 90 s because the authors of [16] previously suggested that adding accommodating resistance is an efficient strategy for achieving PAPE in explosive activities within a short timeframe. The choice of a 90 s rest period between the ERPP and performance assessment was based on their previous study, which compared the effects at 90, 120, and 150 s, finding that 90 s was the optimal rest time [16].

From a practical application standpoint, our data suggest that using resistance bands during a warm-up is an efficient strategy for inducing PAPE, as previous authors reported [44] for individuals with lower strength levels. Therefore, it is highly practical because of the portability of resistance bands and the ability to achieve PAPE more quickly, making it a suitable strategy for optimizing performance in sports. For example, in team sports that allow substitutions, such as basketball, this may serve as an effective potentiation strategy for the transition from rest to competition. Our data provide valuable insights into the application of PAPE protocols using an elastic-resisted push-press for upper- and lower-body exercises and monitoring using RPE-1. However, further research is needed to optimize stimulus selection for inducing PAPE in different athletic populations and movement patterns.

### Limitations and Directions for Further Research

The PAPE phenomenon observed in the less strong participants compared to the stronger participants can be attributed to the instructions given to all the participants to perform the movement with maximum speed intention, influenced by the applied load. The load of the elastic resistance may be underestimated, indicating the need for better familiarization with the perceived rate of exertion in the first repetition (RPE-1).

Additionally, a 90 s rest period was allowed after the PAPE induction stimulus, which may have contributed to the results. This duration might be insufficient for optimal recovery and subsequent performance enhancement. The relatively short rest interval could limit the physiological mechanisms to induce an effective post-activation performance enhancement, potentially impacting the efficacy of the PAPE protocol. Further research is needed to determine the ideal rest duration to maximize PAPE benefits and the potential physiological mechanisms underlying PAPE induced by resistance bands with a short rest period. Therefore, further research is recommended to investigate the underlying mechanisms involved in the induction of PAPE when using resistance bands over short time intervals. Such studies could examine the neuromuscular adaptations, muscle activation patterns, and physiological responses specific to resistance bands, contributing to a deeper understanding of how these variables interact to optimize PAPE. Additionally, comparisons with other forms of resistance, such as free weights or isoinertial devices, would be valuable to clarify the unique effects and potential advantages of resistance bands in inducing PAPE.

## 5. Conclusions

In this pilot study, we found that an elastic resistance push-press (ERPP) induced post-activation performance enhancement (PAPE) in participants with lower strength levels, leading to improvements in mean propulsive velocity (MPV) in push-ups, as well as enhanced countermovement jump (CMJ) and sprint performance over 10 and 20 m. These findings suggest that ERPP may be a practical strategy to acutely induce PAPE in weaker athletes when time or equipment access is limited. Additionally, we propose RPE-1 as a practical tool for monitoring intensity in elastic band resistance exercises to elicit PAPE. Based on these findings, we recommend incorporating ERPP in warm-up or when a substitution should be made for a player on the bench as an efficient and practical strategy for inducing PAPE, with RPE-1 serving as an effective tool for intensity monitoring.

## Figures and Tables

**Table 1:** Push-up performance (m/s); mean ± SD.

	Mean Propulsive Velocity (m/s)		Maximum Speed (m/s)	
	Less Strong (n = 12)	Stronger (n = 12)	Less Strong (n = 12)	Stronger (n = 12)
Control	0.49 (0.17)	0.77 (0.09)	0.95 (0.24)	1.23 (0.22)
ERPP	0.59 (0.13) *	0.77 (0.14)	1.05 (0.26)	1.18 (0.23)
Effect size (Cohen’s d) **	0.66 (0.20–1.52)	0 (−0.8–0.8)	0.4 (−0.41–1.21)	−0.22 (−1.03–0.58)

* Statistical difference between conditions (*p* ≤ 0.05). ERPP: elastic resistance push-press. m/s: meters per second. ** 95% Confidence Interval for Cohen’s d.

**Table 2:** CMJ performance (centimeters); mean ± SD.

	Less Strong (n = 11)	Stronger (n = 13)
Control	31.54 (3.29)	42.08 (4.66)
ERPP	33.46 (4.80) *	41.27 (6.35)
Effect size (Cohen’s d) **	0.47 (0.38–1.31)	−0.15 (−0.98–0.69)

* Trend toward statistical significance between conditions, with a medium effect size (*p* = 0.13). ERPP: elastic resistance push-press. cm: centimeters. ** 95% Confidence Interval for Cohen’s d.

**Table 3:** The time of the 10 m and 20 m sprints (seconds); mean ± SD.

	10 m (sec)		20 m (sec)	
	Less Strong (n = 11)	Stronger (n = 13)	Less Strong (n = 11)	Stronger (n = 13)
Control	2.23 (0.20)	1.93 (0.19)	3.69 (0.24)	3.28 (0.23)
ERPP	2.05 (0.13) *	2.04 (0.17) *	3.48 (0.13) *	3.45 (0.09) *
Effect size (Cohen’s d) **	−1.07 (−1.92–−0.21)	0.61 (−0.21–1.43)	−1.09 (−1.95–−0.23)	0.97 (0.13–1.82)

* Statistical difference (*p* ≤ 0.05). ERPP: elastic resistance push-press. sec: second. ** 95% Confidence Interval for Cohen’s d.

## Data Availability

The dataset is available on request from the authors. The raw data supporting the conclusions of this article will be made available by the authors on request.
